# *Dicyphus cerastii*: First data on development, survival, and reproduction

**DOI:** 10.1371/journal.pone.0320847

**Published:** 2025-04-09

**Authors:** Gonçalo Abraços-Duarte, Filipe Madeira, Paula Souto, Elsa Borges da Silva, Elisabete Figueiredo

**Affiliations:** 1 Instituto Superior de Agronomia, Universidade de Lisboa, Lisboa, Portugal; 2 LEAF, Linking Landscape, Environment, Agriculture and Food, Associate Laboratory TERRA, Lisboa, Portugal; 3 CEF, Forest Research Centre, Associate Laboratory TERRA, Lisboa, Portugal; 4 ESAS, Santarém Polytechnic University, Santarém, Portugal; 5 Research Centre for Natural Resources Environment and Society (CERNAS), Polytechnic Institute of Coimbra, Coimbra, Portugal; 6 Centre for Functional Ecology—Science for People & the Planet (CFE), Department of Life Sciences, Associate Laboratory TERRA, University of Coimbra, Coimbra, Portugal; Manonmaniam Sundaranar University, INDIA

## Abstract

*Dicyphus cerastii* Wagner (Hemiptera: Miridae) is an important predator in horticultural crops. This study provides the first data on biological traits like development, survival, and reproduction for this species. We investigated how host (tomato, tobacco, and Cape gooseberry) and temperature (15.0, 20.0, 25.0 ±  1 °C) influenced nymphal development, survival, and adult longevity. In the absence of prey, nymphs failed to complete development on any host. When prey was available, nymphal development, survival and longevity declined as temperature increased across all hosts. Development and longevity of *D. cerastii* were further examined on tomato, at seven temperatures (15.0, 20.0, 25.0, 27.5, 30.0, 32.5, 35.0 ±  1°C). Reproductive capacity was measured at 20.0, 25.0, 30.0 ±  1°C, on tomato. Egg development ranged from 30.6 days (15.0 °C) to 9.7 days (32.5 °C). Nymph development decreased from 40.0 days (15.0 °C) to 16.4 days (30.0 °C), and no nymphs completed development above 30.0 °C. The optimal temperature for development from egg to adult was estimated at 29.2 °C., while the minimum threshold for immature development was approximately 7.0 °C. The thermal constant for development was 230.4 degree-days for eggs, and 394.0 degree-days for nymphs. Adult longevity ranged from 158.6 days (15.0 °C) to 13.8 days (30.0 °C). The net reproductive rate (R_0_) and generation time (*T*) were highest at 20.0 °C, while the intrinsic rate of increase (*r*_*m*_) was highest at 25.0 °C.

## Introduction

Dicyphine mirids (Miridae: Dicyphini) are important natural enemies on horticultural crops. This tribe includes many zoophytophagous predators essential for biological control in protected crops globally [1–4].

In Europe, important dicyphine predators usually belong to the genera *Dicyphus* Fieber, *Macrolophus* Fieber and *Nesidiocoris* Kirkaldy. Some species like *Macrolophus pygmaeus* (Rambur) and *Nesidiocoris tenuis* (Reuter) are currently mass reared and commercialized for augmentation strategies [5]. Differently, European *Dicyphus* species are mostly regarded in conservation biological control (CBC) [6,7].

In Portugal and Spain, growers resort mostly to commercial releases of *N. tenuis* to control whiteflies (Hemiptera: Aleyrodidae) and *Phthorimaea absoluta* Meyrick (Lepidoptera: Gelechiidae) on protected tomato crops. Although effective in summer, this predator struggles to establish early spring populations and is more termophilous than other European dicyphines [8,9]. Another downside is that despite being a valuable predator, *N. tenuis* can also feed on, and damage tomato plants [1,10]. Because of this behaviour, tomato growers often resort to pesticides to manage *N. tenuis* populations [11], disturbing biological control services provided by other natural enemies. Consequently, there has been an increasing interest in evaluating other dicyphine species that may be an alternative to *N. tenuis* [9,12,13].

*Dicyphus cerastii* Wagner is vastly distributed across the Mediterranean region [14,15]. In Portugal, this species is commonly found in protected tomato crops under low pesticide usage [11,16,17]. This generalist predator feeds on several horticultural pests [16,18–20] and exhibits particularly high predation rates over economically important pests such as *P. absoluta* and *Bemisia tabaci* (Gennadius) (Hemiptera: Aleyrodidae), even when compared with other dicyphines [19]. Being zoophytophagous, *D. cerastii* can also originate plant damage resulting from its phytophagous behaviour, but still less severe compared to *N. tenuis* [21].

*Dicyphus cerastii* used to be the predominant mirid species in Portuguese tomato greenhouses [11,16], but *N. tenuis* has recently become more abundant than *D. cerastii* [11,17]. A seasonal abundance shift has also been observed, with *D. cerastii* populations decreasing during summer whereas those of *N. tenuis* increase [11].

Due to its distribution, predatory efficiency, and lower phytophagy, *D. cerastii* is a promising biological control agent (BCA) for tomato crops [19,21]. This species could, presumably, be an alternative or a complement to *N. tenuis* in Portuguese greenhouses. However, there is only limited information about its life history parameters, and the influence of external factors like host plant and/or temperature on its biology remains largely unknown.

Autochthonous species, that are not currently mass produced, provide services through CBC strategies, which often considers habitat enrichment by providing alternative host plants [22,23]. In dicyphines, host plant species can influence different traits such as predation [24], survival [25], and reproduction [26,27]. Therefore, information on how alternative hosts affect the performance of BCAs, is fundamental to select plants that provide the best conditions for population build up and establishment on crops. Dicyphines are specialists of glandular trichome bearing plants [28], and *D. cerastii* is commonly found on tobacco (*Nicotiana tabacum* L.) and Cape gooseberry (*Physalis peruviana* L.) in gardens (our pers. obs.). To know how these host species influence *D. cerastii*, we compared the post-embryonic period, survival and longevity on tomato, tobacco and Cape gooseberry at 15.0, 20.0 and 25.0 ± 1.0 °C.

As for most insects, temperature plays a determinant role in the biological traits and, consequently, on the geographical distribution of dicyphines. Even co-occurring species display different immature development and thermal activity thresholds, which make them differently adapted for distinct climates or growing seasons [9]. Temperature also influences the predatory capacity of these mirids [29,30], and the damage induced by their phytophagy [31,32]. Therefore, when evaluating new candidate BCA species, like *D. cerastii*, it is determinant to understand how temperature influences its life history parameters. In this context, we examined *D. cerastii* development, survival, and longevity across a wide range of constant temperatures to model its temperature-dependent development rate. Additionally, we assessed its reproductive parameters to determine key demographic traits, providing valuable insights into its potential application as a BCA. This study is the first to examine the life history parameters of *D. cerastii*, a native Mediterranean species with promising potential as a BCA.

## Materials and methods

### Ethics statement

Insects collected from the field were obtained from private properties with owners’ consent. The fieldwork did not involve any endangered or protected species.

### Insects and host plants

*Dicyphus cerastii* was originally collected from different geographical sites in Portugal: Fataca (Odemira, Beja district) (collected from Cape gooseberry and *Pelargonium* spp. in gardens); Ferreira do Zêzere (Santarém district) (collected on garden Cape gooseberry and tomato), Lisbon and Sintra (Lisbon district) (collected on Cape gooseberry and tomato), and Torres Vedras (Lisbon district) (always collected on commercial tomato greenhouses either on tomato or tobacco), and Póvoa de Varzim (Porto district) (from commercial tomato greenhouses on tomato/tobacco). From these populations, a mixed population colony was started, which was frequently refreshed with wild individuals, to prevent lack of genetic diversity.

Rearing units were kept in 40x40x60 cm mesh cages (Entosphinx, Pardubice, Czech Republic) with tobacco plants (ca. 20–30 cm high). These units were fed weekly with a mix of *Artemia* sp. (Anostraca: Artemiidae) cysts and *Ephestia kuehniella* Zeller (Lepidoptera: Pyralidae) eggs (Entofood®, Koppert Biological Systems, Berkel en Rodenrijs, The Netherlands) provided *ad libitum*. Bee pollen (Serramel, Euromel Apicultores, Penamacor, Portugal) was also sprinkled on tobacco leaves at the time of feeding.

To obtain 1^st^ instar nymphs for experiments, *D. cerastii* adults were kept in a cage for 2 weeks for oviposition. After that newly emerging nymphs were collected daily for use in biological assays and to refresh the rearing units.

All rearing units were kept under laboratory conditions in the insectary at Instituto Superior de Agronomia (Lisbon, Portugal), at 25 ±  2 °C, 65 ±  5% h.r. and a 14 h photoperiod.

All plants were grown at ISA’s facilities and were not sprayed with pesticides. Plants were fertilized once a week and watered as needed. To prevent contamination from fungi or arthropods, host plant leaves were washed with abundant water, then bathed in a 5% solution of sodium hypochlorite for 10 min, and rinsed in water again, before being used in bioassays.

#### Nymphal development, survival, and adult longevity on different host plants.

Nymphal development was compared between the host plants tomato, Cape gooseberry and tobacco at three temperatures (15.0, 20.0, 25 ±  1.0 °C). For these experiments, plastic 100 ml Deli cups, with a meshed lid were used. For all three host plants, leaf discs ( ∅ 30 mm) were placed in the Deli cups, abaxial side up, on top of a moistened cotton disc ( ∅ 60 mm). Factitious prey (Entofood®) food was provided on a 2 cm^2^ sticky paper, placed in the cup.

First instar nymphs (<24 h old) were collected from rearing cages and placed individually in a Deli cup. These cups were kept in climatic chambers (Fitoclima S600; Aralab, Rio de Mouro, Portugal) at each of the three selected temperatures. These bioassays were performed in both presence and absence of factitious prey (Entofood®), in which case no sticky paper was added to the cup.

Nymphs were checked every 24 h and the respective instar and mortality were recorded. Every two days, the insects were moved into a new cup, with a new cotton disc, fresh leaf disc, and sticky paper with food. This procedure was maintained until the death of adult insects to determine longevity.

#### Life history parameters on tomato.

Nymphal survival without prey was higher on tomato, and given the economic importance of this crop, life history parameters were further studied on this host in a wider range of temperatures, to allow modeling temperature-dependent development rate.

***Embryonic development on tomato*:** To measure embryonic development, 3–4 week-old tomato plants (*cv* San Pedro, Vilmorin Iberica S.A., Alicante, Spain) were placed in 300 ml plastic cups. A hole was created in the bottom of the cup, and the plant stem was pushed through it. This cup was then placed in a smaller (200 ml) cup which had water accessible only to the roots. In each cup, factitious prey (Entofood®) was provided on a 2 cm^2^ sticky paper. Plastic cups were placed for 24 h in a mesh cage (35x35x35 cm) (Entosphinx, Pardubice, Czech Republic) containing ca. 50 adult couples of *D. cerastii*. After this period, adult mirids were removed, and the cups were placed in climatic chambers at different temperatures (15.0, 20.0, 25.0, 27.5, 30.0, 32.5, 35.0 ±  1.0 °C) and were daily inspected to record the number of nymphs emerging.

***Post-embryonic development and adult longevity on tomato*:** Development was observed on tomato *cv* San Pedro at the constant temperatures of 15.0, 20.0, 25.0, 27.5, 30.0, 32.5, and 35.0 ±  1.0 °C. These bioassays followed the same procedure as previously described for different host plants, except that, in this experiment, the Deli cups at higher temperatures (27.5, 30.0, 32.5, and 35.0 °C) were changed every other day as the cotton disc would dry too quickly.

***Reproduction on tomato*:** Reproductive parameters were observed on tomato at three different temperatures (20.0, 25.0 and 30 ±  1.0 °C). Adult couples used in these experiments were obtained by placing first instar nymphs ( < 24 h old) in identical conditions to those previously described for rearing units, in climatic chambers at each of the tested temperatures (20.0, 25.0 and 30.0 ±  1.0 °C), 60 ±  10% r.h., and 14 h photoperiod. The cages were observed daily and emerging adult couples ( < 24 h old) were collected and placed in plastic cups (identical to those used in embryonic development bioassays) with a tomato plant cv San Pedro and a 2 cm^2^ sticky paper with factitious prey (Entofood ®). The couples were daily moved into new cups (with new plant and fresh prey) until nymphs started to emerge, to record the pre-oviposition period. After this, couples were moved into new cups every three days until female death. Males that died during experiments were replaced with others from the respective temperature. At least 20 couples were tested for each temperature.

The eggs of dicyphines are laid inside plant tissue and difficult to count. In preliminary experiments, we observed that the number of emerged nymphs was often higher than that of counted eggs. Therefore, in this work, we used the number of emerged nymphs rather than eggs to infer the fertility of *D. cerastii*. Because of this, it must also be noted that the pre-oviposition period we recorded corresponds to the period before the emergence of the first nymphs and not of the first eggs laid.

#### Data analysis.

For the comparison of different host plants, the duration of nymphal development and adult longevity were calculated. These response variables were analyzed using Generalized Linear Models (GLM) based on a Gaussian distribution with an identity link function. The data were not transformed, and normal diagnostic checks were conducted to assess the normality of residuals and homoscedasticity, to verify these assumptions (Shapiro-Wilk Test, Residuals vs Fitted Values Plot, Histogram of Residuals and Q-Q Plot). For each of the response variables, the explanatory variables in the model were ‘host’ (tomato, Cape gooseberry, tobacco), ‘temperature’ (15.0 °C, 20.0 °C and 25.0 °C) and ‘sex’ (male and female), and their interactions. The models were simplified with a stepwise model selection based on the Akaike information criterion (AIC) using the ‘stepAIC’ function of the *MASS* package in the R software [33]. When significant differences were detected, post-hoc comparisons of mean values were performed with the Tukey HSD method, in the *agricolae* R package [34].

All survival curves of *D. cerastii* at each temperature and host were estimated using the Kaplan-Meier method. Log-rank tests were used to compare survival curves. For this, the R packages *survival* [35] and *survminer* [36] were used.

For tomato, the duration of egg, each nymphal instar, total nymph development time and adult longevity were calculated for all the temperatures that allowed completion of immature development. These response variables were also analyzed using GLM based on a Gaussian distribution with an identity link function. For egg development the explanatory variable in the model was ‘temperature’ (15.0, 20.0, 25.0, 27.5, 30.0, 32.5 °C). For nymphal instar, post-embryonic development time and adult longevity the explanatory variables in the model were ‘temperature’ (15.0, 20.0, 25.0, 27.5, 30.0 °C) and ‘sex’ (male, female), and their interactions. As previously described, models were simplified with a stepwise model selection based on the AIC. The Tukey HSD method was used to perform post-hoc mean values comparisons when significant differences were detected.

To describe the temperature-dependent developmental rate relationship, three mathematical models were used, a linear model [37] and two non-linear ones (Lactin-2, Brière-1) [38,39]. Lower, optimal and upper temperatures were obtained for all immature stages. The two non-linear models were chosen since they are commonly used for modeling insect developmental rates [40,41] having been used for Heteroptera [40,42,43]. Only the non-linear models allowed calculating the optimum temperature (t_*opt*_) and the maximum (t_*max*_) developmental threshold, whereas for all the models the lower developmental threshold (t_*min*_) was estimated as the value intercepting the temperature axis. The goodness of fit was evaluated by the coefficient of determination for both linear and non-linear models (R^2^, higher value indicated better fitting), the residual sum of squares (RSS, lower value indicated better fitting), the AIC (lower value indicated better fitting), and by biological criteria. Data was fitted using the *devRate* R package [44]. Initial parameter estimation for the Lactin-2 model was made following the suggestions of Logan [45]. All statistical analyses referring to model adjustment and comparisons were performed using R version 3.5.2 [46]. The thermal constant (K) of each immature stage of *D. cerastii* was also calculated; this constant was estimated using the linear model as the reciprocal of the slope b (K = 1/b). To adjust the linear and Brière-1 models, last data values, 32.5 °C and 35.0 °C, were omitted. This was necessary for the correct calculation of the parameters K and t_*min*_ in the case of the linear model [47] and to estimate the lower developmental threshold (t_*min*_), the optimum temperatures (t_*opt*_), and the maximum lethal temperature (t_*max*_) for the Brière-1 model. The equations of the linear and each of the two non-linear models are detailed in S1 Table in S1 File.

Finally, for tomato, a life table was built with data from immature development and reproduction bioassays at 20 °C and 25 °C, but not from 30 °C since fertility was very low. Sex ratio, adult daily survival, the pre-oviposition time, and the number of offspring produced by the females were recorded. The net reproductive rate (*R*_*0*_; female offspring per female), generation time (*T*; days), the intrinsic rate of increase (*r*_*m*_; females per female per day), doubling time (*DT*; days), and the finite rate of increase (*ʎ*; females per female per day) were calculated according to Birch [48]. The standard error associated with *R*_*0*_, *T*, *r*_*m*_, *DT* and *ʎ* was estimated by bootstrapping (100,000 replications). To compare differences between temperatures, paired bootstrap tests were used (α =  0.05).

## Results

### Nymphal development, survival, and longevity on different host plants

**Fig 1:**
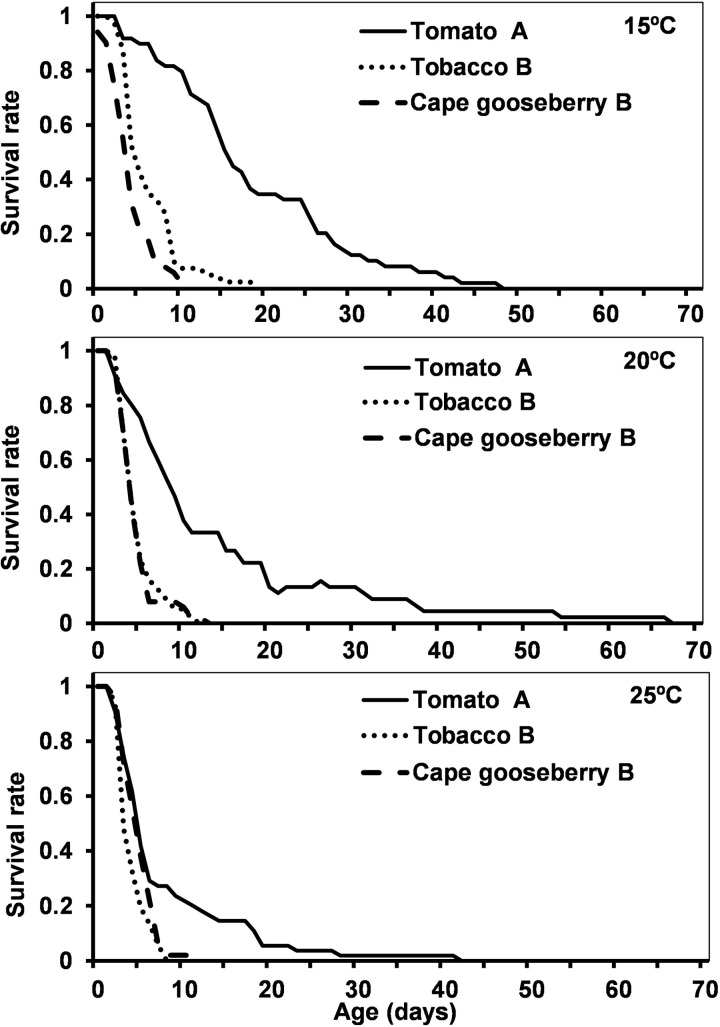
Survival (days) of *Dicyphus cerastii* nymphs on three plant hosts (tomato, tobacco, Cape gooseberry) at three different temperatures (15.0, 20.0, 25.0 ± 1.0 °C) without factitious prey. For each temperature, different letters in front of host species correspond to significantly different survival curves for Log-Rank comparison test (p < 0.05).

In absence of factitious prey, *D. cerastii* nymphs were unable to complete development on all host plants. Nymphs survived longer on tomato compared to Cape gooseberry and tobacco (Table 1, Fig 1). On tomato, some nymphs could reach the 5^th^ instar, whereas on Cape gooseberry none could complete the 2^nd^ instar, and on tobacco one nymph reached the 3^rd^ instar but didn’t complete it. Temperature also influenced nymph survival on each host differently: on tomato survival decreased only at 25 °C; on Cape gooseberry, survival decreased at 20 °C and was similar at 25 °C; on tobacco, survival decreased as temperature increased (Table 1, Fig 1, S2 Table in S1 File).

**Table 1:** Survival in days (mean ±  SE) of *Dicyphus cerastii* at three different temperatures (15.0, 20.0, 25.0 ±  1.0 °C) on three different hosts (tomato, Cape gooseberry, tobacco), both without and with factitious prey.

Host	Without factitious prey			With factitious prey		
	15 °C	20 °C	25 °C	15 °C	20 °C	25 °C
Tomato	17.9 ± 1.7aA (46)	13.3 ± 2.1aA (44)	6.8 ± 1.0bA (54)	198.6 ± 11.8aAB (50)	96.1 ± 8.0bAB (52)	55.7 ± 3.8cA (55)
Cape gooseberry	5.1 ± 0.4aB (51)	3.7 ± 0.3bB (50)	4.0 ± 0.3bB (49)	218.6 ± 10.2aA (52)	103.2 ± 6.3bA (55)	66.5 ± 4.4cAB (50)
Tobacco	5.3 ± 0.6aB (41)	3.8 ± 0.3bB (54)	3.1 ± 0.2cB (52)	194.9 ± 23.1aB (48)	103.0 ± 6.2aB (48)	70.4 ± 4.7bB (39)

Means followed by different lowercase letters within rows (a, b, c), or uppercase within columns (A, B), correspond to groups among which survival curves are significantly different for Log-Rank comparison test (*p* < 0.05) (within treatments: without or with factitious prey). Values within brackets are the number of nymphs used in each combination of host, prey availability and temperature.

**Fig 2:**
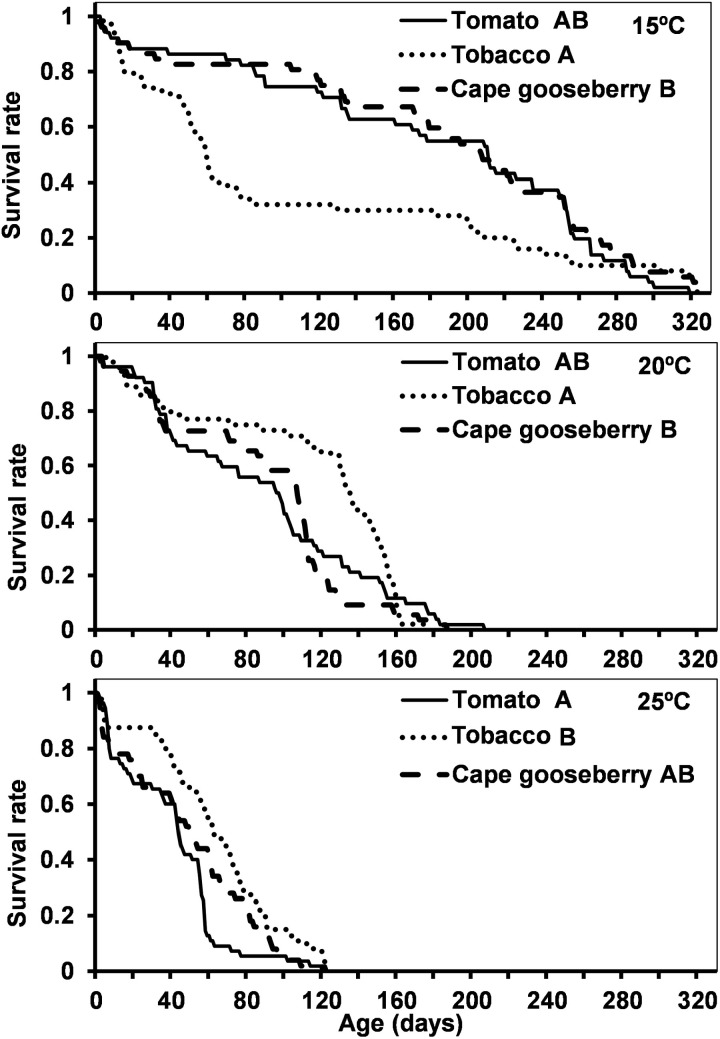
Survival (days) of *Dicyphus cerastii* on three plant hosts (tomato, tobacco, Cape gooseberry) at three different temperatures (15.0, 20.0, 25.0 ±  1.0 °C) with factitious prey. For each temperature, different letters in front of host species correspond to significantly different survival curves for Log-Rank comparison test (p < 0.05).

In the presence of factitious prey, survival decreased with increasing temperature, on all hosts. The longest survival was observed on Cape gooseberry at 15.0 °C and the shortest on tomato at 25.0 °C (Table 1, Fig 2, S3 Table in S1 File).

In the presence of prey, nymph mortality was generally under 20% (except for tobacco at 15.0 °C, and tomato and Cape gooseberry at 25.0 °C) (Table 2). Host, temperature, and their interaction influenced the developmental period of *D. cerastii* nymphs, whereas sex did not. On all hosts, post-embryonic development time decreased with increasing temperature. At 15.0 °C development was fastest on tomato, and no differences were observed between Cape gooseberry and tobacco; at 20.0 °C there were no differences between hosts; and at 25.0 °C development time was shortest on tobacco (Table 2, S4 Table in S1 File).

**Table 2:** Duration in days (mean ±  SE) of post-embryonic development, adult longevity, and percentage of nymph mortality of *Dicyphus cerastii* at three different temperatures (15.0, 20.0, 25.0 ±  1.0 °C) on three different hosts (tomato, Cape gooseberry, tobacco) with factitious prey.

Host	15.0 °C			20.0 °C			25.0 °C		
	Development (days)	Nymph mortality (%)	Longevity (days)	Development (days)	Nymph mortality (%)	Longevity (days)	Development (days)	Nymph mortality (%)	Longevity (days)
Tomato	40.0 ± 0.5aA	12.0 (50)	158.6 ± 12.0aA	25.1 ± 0.3bA	7.7 (52)	70.9 ± 8.2bA	20.0 ± 0.3cA	32.7 (55)	35.7 ± 4.0cA
Cape gooseberry	44.7 ± 0.6aB	17.3 (52)	173.7 ± 10.4aA	25.7 ± 0.5bA	14.5 (55)	77.2 ± 6.4bA	19.6 ± 0.6cAB	26.0 (50)	46.7 ± 4.5cAB
Tobacco	45.2 ± 0.7aB	54.2 (48)	149.2 ± 23.8aA	25.8 ± 0.4bA	16.7 (48)	106.3 ± 6.4bB	18.6 ± 0.3cB	10.3 (39)	51.7 ± 4.8cB

Means followed by the same lowercase letter within rows, or uppercase within columns, correspond to groups (within each response variable) among which means are not significantly different for Tukey HSD test (p < 0.05). Values within brackets are the number of nymphs used in each combination of host and temperature.

Adult longevity was not significantly influenced by the host alone (S4 Table in S1 File). However, temperature, sex, and the interactions of the host with temperature, and sex with temperature were significant (Table 2, S4 Table in S1 File). Both male and female longevity decreased with increasing temperature. At 15.0 °C and 25.0 °C male longevity was higher than that of females, whereas at 20.0 °C there were no significant differences (S5 Table in S1 File).

### Life history parameters on tomato

#### Embryonic and post-embryonic development, longevity, and survival on tomato.

Eggs hatched at all temperatures except at 35.0 °C. Embryonic development was influenced by temperature and was longest at 15.0 °C and shortest at 30.0 °C, however there were no significant differences between 27.5 °C, 30.0 °C and 32.5 °C (Table 3, S6 Table in S1 File).

**Table 3:** Duration in days (mean ±  SE) of the egg, nymphal instars, total nymphal development, adult longevity, percentage of nymphal mortality, and sex ratio (proportion of females) of *Dicyphus cerastii* at seven different temperatures (15.0, 20.0, 25.0, 27.5, 30.0, 32.5, 35.0 ±  1.0 °C) on tomato with factitious prey.

T °C	Egg	Instar					Nymph mortality (%)	Total	Sex ratio	Longevity
		1^st^	2^nd^	3^rd^	4^th^	5^th^				
15.0 °C	30.6 ± 0.2a (204)	7.5 ± 0.2a	6.4 ± 0.1a	6.2 ± 0.2a	7.4 ± 0.2a	12.6 ± 0.2a	12.0ab (50)	40.0 ± 0.5a	0.32	158.6 ± 12.0a
20.0 °C	16.5 ± 0.1b (111)	4.5 ± 0.1b	3.7 ± 0.1b	3.7 ± 0.1b	4.7 ± 0.1b	8.0 ± 0.1b	7.7a (52)	25.1 ± 0.3b	0.60	70.9 ± 8.2b
25.0 °C	11.2 ± 0.1c (98)	4.1 ± 0.1b	2.8 ± 0.1c	3.0 ± 0.1c	3.6 ± 0.1c	6.5 ± 0.1c	32.7ce (55)	20.0 ± 0.3c	0.57	35.7 ± 4.0c
27.5 °C	9.8 ± 0.1d (88)	3.0 ± 0.1c	2.3 ± 0.1c	2.5 ± 0.1c	3.2 ± 0.1c	5.0 ± 0.1d	25.0bc (52)	16.1 ± 0.2d	0.51	12.4 ± 1.1c
30.0 °C	9.6 ± 0.1d (49)	3.1 ± 0.1c	2.7 ± 0.1c	2.7 ± 0.1c	3.3 ± 0.1c	4.6 ± 0.2d	51.9de (54)	16.4 ± 0.3d	0.42	13.8 ± 1.6c
32.5 °C	9.7 ± 0.1d (49)	n.d.	n.d.	n.d.	n.d.	n.d.	100.0^*^ (52)	n.d.		
35.0 °C	n.d.	n.d.	n.d.	n.d.	n.d.	n.d.	100.0^*^ (40)	n.d.		

Means followed by the same letter within columns correspond to groups among which values are not significantly different for Tukey HSD multiple comparison test (p < 0.05). Nymph mortality percentages, followed by the same letter within columns, correspond to groups among which survival curves are not significantly different for Log-Rank comparison test (p < 0.05).

* - not considered in the analysis; n.d. – no development. Values within brackets are the number of hatched eggs and nymphs used in each temperature.

As observed above, for host comparison (Cape gooseberry, tobacco), sex had no influence on post-embryonic development time (S6 Table in S1 File), which decreased with increasing temperature, but there were no significant differences between 27.5 °C and 30.0 °C (Table 3). *Dicyphus cerastii* could not complete development at 32.5 °C and 35.0 °C. In fact, at 32.5 °C some individuals were able to reach the 3^rd^ instar whereas at 35.0 °C only three individuals were able to complete the 1^st^ instar.

**Fig 3:**
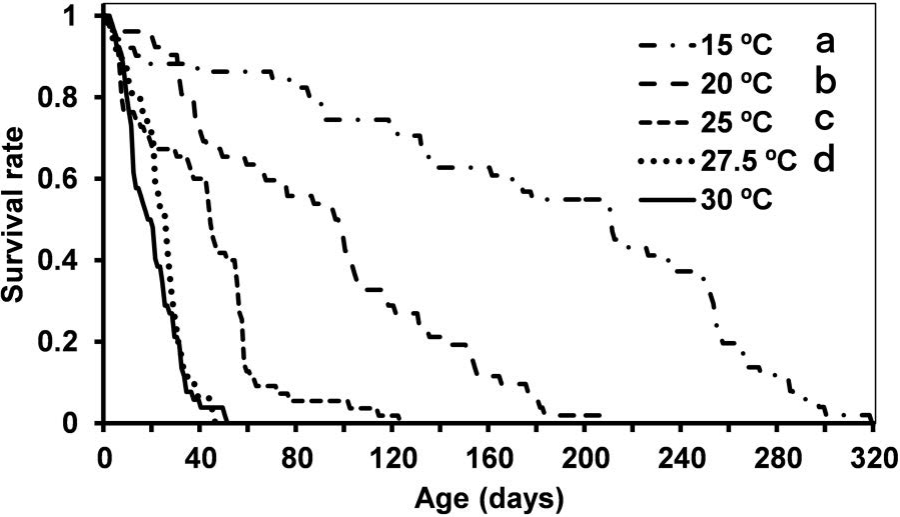
Survival (days) of *Dicyphus cerastii* on tomato at five different temperatures (15.0, 20.0, 25.0, 27.5 and 30.0 ±  1.0 °C) with factitious prey. Different letters in front of each temperature correspond to significantly different survival curves for Log-Rank comparison test (p < 0.05).

Nymph mortality varied among temperatures. At 15.0 °C and 20.0 °C more nymphs were able to complete development compared to the other temperatures. Among the temperatures that allowed development, the lowest survival was recorded at 30.0 °C (Table 3, S7 Table in S1 File). Overall survival curves (from hatching to adult death) differed among all temperatures, except between 27.5 °C and 30 °C (Fig 3, S8 Table in S1 File).

Temperature also influenced adult longevity, whereas sex did not. Longevity was highest at 15.0 °C, followed by 20.0 °C, and there were no significant differences between 25.0 °C, 27.5 °C and 30.0 °C (Table 3, S6 Table in S1 File).

All the three models used provided a good fit to the relationship between temperature and the development rates of egg, each nymphal instar, post-embryonic or total immature (S9 Table in S1 File). Despite this, the Brière-1 model provided negative minimum thresholds (t_*min*_) for the first and fifth nymphal instar, and for total post-embryonic development. Fitting the Linear model presented lower minimum development thresholds (t_*min*_) compared to the Lactin-2 model for both post-embryonic (4.74 and 6.00 °C, respectively) and total immature (6.26 and 7.50 °C, respectively). The Lactin-2 model estimated the optimal temperature for total development (t_*opt*_) to be at 29.20 °C. The thermal constant K values were 230.36 degree-days (DD) for eggs, 393.98 DD for nymphs, and 584.76 DD for total egg to adult development (S9 Table in S1 File).

#### Reproduction and demographic parameters.

At 20.0 °C, individual *D. cerastii* females produced 159.6 ±  23.7 (n = 18) nymphs during their 87.8 ±  6.5 days lifespan, while at 25.0 °C they generated 116.5 ±  15.0 (n = 20) nymphs in their 41.6 ±  3.8 days lifespan, but there were no significant differences between the number of nymphs produced (U = 141.5, p = 0.260). These values resulted in a fertility rate of 2.1 ±  0.3 and 3.7 ±  0.4 nymphs per female per day at 20 °C and 25 °C, respectively, which were significantly different (U = 74.0, *p* = 0.002). Couples kept at 30.0 °C produced very few nymphs (only 3 nymphs were obtained from 21 couples).

**Fig 4:**
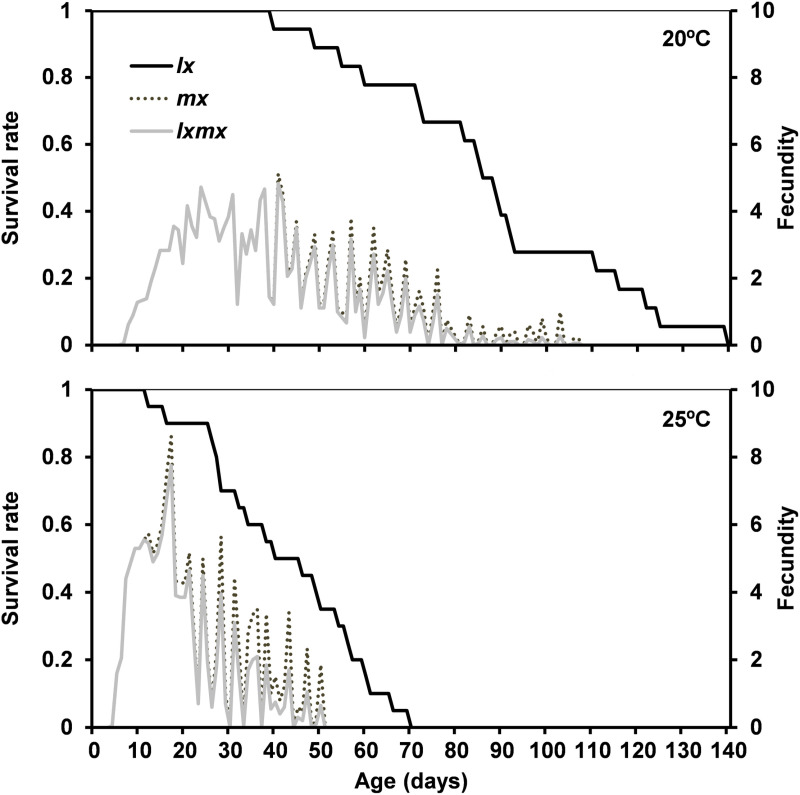
Age-specific survival rate (*lx*), age-specific fertility (*mx*) and age-specific net maternity (*lxmx*) of *Dicyphus cerastii* females at 20.0 °C and 25.0±  1.0 °C on tomato with factitious prey.

Temperature influenced the demographic parameters. The pre-oviposition (emergence of the first nymphs) as well as the oviposition period were longer at 20 °C compared to 25 °C (Fig 4, Table 4). The net reproductive rate (*R*_*0*_), mean generation time (*T)*, and doubling time (*DT*) of *D. cerastii* were higher at 20.0 °C whereas the intrinsic rate of increase (*r*_*m*_), and the finite rate of increase (*λ*) were higher at 25.0 °C (Table 4).

**Table 4:** Mean (±SE) pre-oviposition time (days), net reproductive rate (*R*_*0*_, female offspring per female), mean generation time (*T*, days), intrinsic rate of increase (*r*_*m*_, day^-1^) doubling time (*DT*, days) and finite rate of increase (*λ*, day^-1^) of *D. cerastii* on tomato with factitious prey at 20.0 °C and 25.0 ±  1.0 °C.

	Temperature		
Demographic parameters	20.0 °C	25.0 °C	*p*
Pre-oviposition time	17.3 ± 2.0	8.6 ± 1.5	0.0026
*R₀*	88.96 ± 0.41	44.24 ± 0.18	0.0039
*T*	62.85 ± 0.05	39.03 ± 0.08	0.0402
*r*_*m*_	0.067 ± 0.000	0.091 ± 0.000	0.0008
*DT*	10.91 ± 0.23	8.20 ± 0.018	0.0108
*λ*	1.07 ± 0.0001	1.10 ± 0.00	0.0007

SE was estimated by bootstrapping (100.000 replications). p =  P-value calculated using paired bootstrap tests.

## Discussion

The performance of zoophytophagous dicyphines is largely dependent on the availability of prey. Several studies report that when animal prey is absent, nymphs are either unable to complete development or display lower survival rates [12,25,26,49–52]. In the present study, *D. cerastii* was also unable to complete development in the absence of prey on all the hosts tested.

Without prey, *D. cerastii* nymphs survived longer on tomato at all the tested temperatures. A similar trend was reported for *N. tenuis* that survived longer on tomato compared to eggplant and pepper, in absence of prey [25]. Contrary to these results, the predator *Dicyphus hesperus* Knight (Heteroptera: Miridae) survived longer on tobacco than on tomato in the absence of prey [50]. Plant host species can influence survival, development, and performance in dicyphines [25,50,53,54], therefore, *D. cerastii* nymphs may have obtained better nutrition from tomato, over tobacco and Cape gooseberry.

Besides species, other plant attributes influence the performance of dicyphine mirids, such as cultivar [32] and organs. For example, *Dicyphus tamaninii* Wagner (Heteroptera: Miridae), in the absence of prey, can complete development while feeding on tomato fruits but not on leaves [55]. *Nesidiocoris tenuis* also shows better developmental success when nymphs are reared on whole tomato plants compared to excised leaves [56]. Pollen and nectar also have nutritional value to dicyphines [49,52,57,58] and *D. cerastii* may also benefit from floral resources as it is observed feeding on flowers of tobacco and tomato, and recently, also on those of *Calendula officinalis* L., *Fagopyrum esculentum* Moench, and *Phacelia tanacetifolia* Benth. (our pers. obs.). Therefore, though our results suggest that *D. cerastii* may tend to the zoophagous side of the zoophytophagous spectrum, the effect of more complete plant resources on its performance and development in the absence of prey should be investigated in future research.

Development time decreased with increasing temperature, as observed in other studies [9,43,49]. At 15.0 °C, *D. cerastii* nymphs completed development in approximately 40 days, shorter than the 55.9 days required for *N. tenuis* nymphs [8], but similar to other dicyphines like *M. pygmaeus*, *D. eckerleini* Wagner and *D. errans* Wolff, which take 42–43 days [9]. At 20.0 °C there were no differences among hosts, and *D. cerastii* took 25.1 days to complete development on tomato. At this temperature it is already surpassed by *N. tenuis* and *M. pygmaeus* that require only 21.2 and 22 days, respectively [8,9]. However, at 20.0 °C, *D. cerastii* remains similar to *D. eckerleini* and *D. errans* (25–26 days) but faster than *Dicyphus bolivari* Lindberg (28 days) and *Dicyphus flavoviridis* Tamanini (37 days) [9]. At 25.0 °C development was faster on tobacco (18.6 days) than on tomato (20.0 days) but neither were significantly different from Cape gooseberry (19.6 days). The difference between *D. cerastii* and *N. tenuis* further increases at 25.0 °C since the latter needs only about 13 days [8,9]. At this temperature, and depending on the host, *D. cerastii* can be slower or similar to *M. pygmaeus* (ca. 17 days) [9,59], *D. eckerleini* (16 days), *D. errans* (16.3–17 days) [9,60], and *D. bolivari* (ca.19 days) [9,60]. The nymphs of *D. cerastii* took 16.4 days to complete development at 30.0 °C and had a high mortality rate (51.9%). At this temperature, *N. tenuis* needs only about 9 days [8,9], *M. pygmaeus* and *D. errans* are also faster requiring just 13 days [9]. However, *D. cerastii* was faster than *D. bolivari* that needs 18 days [9]. At 35.0 °C *D. cerastii* was unable to complete development, similarly to several other European dicyphine species that cannot tolerate this temperature, with the exception of *M. pygmaeus* depending on the host, as well as *D. bolivari* (Barcelona strain), and *N. tenuis* [8,9,59,61].

Adult longevity was also reduced with increasing temperature. At 15.0 °C, longevity was similar among hosts. At 20.0 °C it was highest on tobacco, and at 25.0 °C it was also highest on tobacco but not significantly different from Cape gooseberry. Differences in longevity between host plants have also been reported in other mirid species [50,62]. Males of *D. cerastii* exhibited higher longevity than females at all temperatures. However, at 20.0 °C this difference was not significant. Other dicyphine males like those of *M. pygmaeus* and *M. costalis* also live longer than females [63,64]. By contrast, there were no differences in longevity between sexes, when tomato was further studied at a wider range of temperatures.

As expected, embryonic development was also faster as temperature increased. *Dicyphus cerastii* eggs didn’t hatch at 35.0 °C, whereas eggs of *N. tenuis* can still hatch at this temperature [8,61]. At 32.5 °C, *D. cerastii* eggs hatched but no nymph completed development. This suggests that *D. cerastii* eggs may be more suited to survive extreme conditions derived from high temperatures than young nymphs, as dicyphine eggs are protected within plant tissues.

The minimum developmental thermal threshold estimated by the Lactin-2 model for *D. cerastii* eggs was higher (8.4 °C) than that of the linear model (6.1 °C), the latter being similar to *M. pygmaeus* (6.9 °C), but lower than *N. tenuis* (9.8–12.1 °C), *M. caliginosus* (8.7 °C) and *D. hesperus* (7.3 °C) [8,59,65–68]. Similarly, the Lactin-2 model also presented a higher threshold for nymphal development (6.0 °C) than the linear model (4.7 °C) for *D. cerastii*. In both cases these values were lower than what is reported for other species like *M. pygmaeus* (9.2 °C), *N. tenuis* (11.7 °C), *M. caliginosus* (7.2 °C) and *D. hesperus* (7.8–8.4 °C) [8,59,65,66]. For total development (egg to adult) the Lactin-2 model estimated 7.5 °C whereas the Linear model estimated 6.26 °C. This was also lower than what is reported for other dicyphine species such as *M. pygmaeus* (8.8 °C), *M. caliginosus* (7.7 °C), and *N. tenuis* (10.9 °C) [59,65,68].

The thermal constant K for *D. cerastii* eggs (230.36 DD) was higher than that described for *M. pygmaeus* (182 DD), *M. caliginosus* (184.8 DD), and *N. tenuis* (148.6 DD) [8,59,65], but identical to that of *D. hesperus* (ca. 230 DD) [66]. Nymphs of *D. cerastii* (393.98 DD) also have higher thermal requirements than *M. pygmaeus* (253 DD), *M. caliginosus* (270.3 DD), *D. hesperus* (274.4–301.9 DD), and *N. tenuis* (182.3 DD) [8,59,65,66]. The same trend was found for egg to adult development, since *D. cerastii* (584.76 DD) showed higher needs than *M. pygmaeus* (431 DD), *M. caliginosus* (495 DD), and *N. tenuis* (318.4 DD) [59,65,68].

Females of *D. cerastii* produced 116.5 nymphs in their lifetime at 25.0 °C, which was more than other dicyphines like *D. maroccanus* (Syn. *D. bolivari*) (50.8), *M. pygmaeus* (≈45–48.1), *N. tenuis* (60.0–83.7), *Campyloneuropsis infumatus* (Carvalho) (81.3), but similar to *Engytatus varians* (Distant) (106.9), and *M. basicornis* (124.1) [12,69–72]. *Dicyphus cerastii* daily fertility at 20.0 °C (2.1 nymphs/day) was lower than at 25 °C (3.7 nymphs/day), which was lower than *N. tenuis* (4.3 nymphs/day) but similar to *M. pygmaeus* (3.1 nymphs/day) and *D. maroccanus* (3.6 nymphs/day) [12,72]. At 30.0 °C, very few nymphs were obtained (3 nymphs from 21 couples). Despite this, in the embryonic development experiment, eggs (laid at 25.0 °C) still hatched when placed at 30.0 °C and even 32.5 °C. This suggests that *D. cerastii* adults obtained from nymphs reared at 30.0 °C suffered a negative impact on their reproductive capacity. By contrast, *N. tenuis* can reproduce at 30.0 °C and even 35.0 °C [8].

Temperature had a clear influence on the demographic parameters of *D. cerastii*. The net reproductive rate, generation, and doubling time were all higher at 20.0 °C, whereas the intrinsic and finite rate of increase were higher at 25.0 °C. At 20.0 °C, *D. cerastii* had a net reproductive rate of 88.96 female nymphs per female, which was lower than *M. pygmaeus* (97.05) at the same temperature [63]. At 25.0 °C, the net reproductive rate of *D. cerastii* was lower (44.24) than at 20.0 °C. However, it was higher than *D. maroccanus* (34.52), *M. pygmaeus* (20.03) and *N. tenuis* (32.21) but similar to *Tupiocoris cucurbitaceus* (Spinola) (46.89) [12,72,73].

The intrinsic rate of increase of *D. cerastii* increased from 0.067 to 0.091 females per female per day at 20.0 °C and 25.0 °C, respectively. *Dicyphus cerastii* increases its population at a similar rate than *M. pygmaeus* at 20.0 °C (*r*_*m*_ = 0.065), and at 25.0 °C it may be similar, if not faster, than *M. pygmaeus*, depending on the diet of this predator (*r*_*m*_ = 0.072–0.097) [63,72]. At 25.0 °C *D. cerastii* increases faster than *D. maroccanus* (*r*_*m*_ = 0.087) but slower than *N. tenuis* (*r*_*m*_ = 0.112) [12,72]. In fact, even in absence of prey and reared on sesame (*Sesamum indicum* L.), *N. tenuis* can display a similar intrinsic rate of increase (*r*_*m*_ =  0.094) to what we found for *D. cerastii* at this temperature [53]. At 20.0 °C, *D. cerastii* takes 68.85 days between two generations whereas *M. pygmaeus* needs 84.50 days [63]. At 25.0 °C the generation time of *D. cerastii* is 39.03 days, whereas *N. tenuis* needs only 31.77 days, and on sesame without prey it still takes just 37.80 days [53,72]. *Dicyphus maroccanus* and *M. pygmaeus* take 40.48 and 40.31 days, respectively [12,72], which is slightly more than *D. cerastii*. Besides temperature, the demographic parameters of dicyphines also depend on the diet [73,74], plant host [53], or a combination of both [63]. Therefore, their impact should also be considered in future research on *D. cerastii*, as this may influence both field performance and mass rearing of this predator.

To our knowledge this is the first study to explore the life history parameters of *D. cerastii*. Our results showed that, despite significant differences on some biological traits, the host species we compared did not differ notably when prey is available. Possibly because all the studied hosts belong to the family Solanaceae. Our results also demonstrate that, in general, *D. cerastii* is outperformed by *N. tenuis* on most of the parameters we observed. This underperformance may be driven, primarily, by its slower development and higher mortality at higher temperatures (above 20.0 °C). We also acknowledge that the diet used in this study (a mix of *E. kuehniella* eggs and *Artemia* sp. cysts) may be considered less optimal than a diet consisting purely of *E. kuehniella* eggs. This could negatively influence the performance of *D. cerastii*. However, the high fertility observed in comparison to other dicyphines suggests otherwise.

Our results indicate that *D. cerastii* is less thermophilous than *N. tenuis*, and could be more adapted to temperate climates, as proposed for other *Dicyphus* species, such as *D. errans* and *D. eckerleini* which have relatively similar development rates as *D. cerastii* [9]. Therefore, *D. cerastii* may be an interesting BCA for cooler conditions, such as those found in early spring in Mediterranean protected tomato crops, when *N. tenuis* does not perform well.

Moreover, *D. cerastii* may complement *N. tenuis* since it also feeds on different horticultural pests [16,18–20] and displays higher predation rate on important pests like *P. absoluta* and *B. tabaci* compared to *N. tenuis* [19]. The presence of *D. cerastii* in early crops may not mean an increased risk to the crop since, despite its ability to induce plant damage, its phytophagous behaviour is less severe compared to *N. tenuis* [21].

Direct interactions between *D. cerastii* and *N. tenuis* have been demonstrated to favor *D. cerastii* [75,76]. Despite this, *D. cerastii*, having a slower population increase rate, may be negatively affected from the outcome of indirect interactions with *N. tenuis*, such as competition for food sources or space. In fact, *N. tenuis* is reported as being able to outcompete other dicyphines like *D. maroccanus* and *M. pygmaeus* [77,78].

Conservation strategies could be key to compensate for the slower population growth of *D. cerastii* and its likely lower capacity to compete with *N. tenuis*. It has been demonstrated that providing additional refuge and food sources between crop cycles allows local production, increased biodiversity, and better establishment of dicyphines on horticultural crops [22,23,79]. Therefore, future research on *D. cerastii* should also focus on its conservation considering a wider range of host species to determine the best strategy to promote the presence and establishment of this BCA in protected crops.

## Supporting information

S1 File **S1 Table.** Modelling mathematical equations used to fit temperature (T) and developmental rate (1/D) relationship. **S2 Table.** Log-Rank test comparison of *Dicyphus cerastii* survival curves on tomato, tobacco, and Cape gooseberry at different temperatures, without factitious prey. **S3 Table.** Log-Rank test comparison of *Dicyphus cerastii* survival curves on tomato, tobacco, and Cape gooseberry at different temperatures, with factitious prey. **S4 Table**. Generalized linear models (GLM) analysis of the effect of the explanatory variables “host”, “temperature” and “sex” on the response variables “Post-embryonic development” and “adult longevity”, of *Dicyphus cerastii* reared on tomato, Cape gooseberry and tobacco at 15, 20 and 25°C, with factitious prey. **S5 Table.** Longevity in days (mean ± SE) of male and female *Dicyphus cerastii* at three different temperatures (15, 20, 25°C) with factitious prey. **S6 Table.** Generalized linear models (GLM) analysis of the effect of the explanatory variables “temperature” and “sex” on the response variables “embryonic development”, “Nymph instar”, “Post-embryonic development” and “adult longevity” of *Dicyphus cerastii* reared on tomato, at 15.0, 20.0, 25.0, 27.5, 30.0 and 32.5°C, with factitious prey. **S7 Table.** Log-Rank test comparison of *Dicyphus cerastii* nymph survival curves on tomato at different temperatures, with factitious prey. **S8 Table.** Log-Rank test comparison of *Dicyphus cerastii* survival curves (from egg hatch to adult death) on tomato at different temperatures, with factitious prey. **S9 Table.** Mean values (± SE) of parameters of three models describing the developmental rate of *Dicyphus cerastii.*
(DOCX)
