# Supplementary material for: Dicyphus cerastii: First data on development, survival, and reproduction
Source: PLoS One. 2025 Apr 9;20(4):e0320847. doi: 10.1371/journal.pone.0320847 (PMC11981183; doi:10.1371/journal.pone.0320847)
Supplement: S1 File — S1 Table. Modelling mathematical equations used to fit temperature (T) and developmental rate (1/D) relationship. S2 Table. Log-Rank test comparison of Dicyphus cerastii survival curves on tomato, tobacco, and Cape gooseberry at different temperatures, without factitious prey. S3 Table. Log-Rank test comparison of Dicyphus cerastii survival curves on tomato, tobacco, and Cape gooseberry at different temperatures, with factitious prey. S4 Table. Generalized linear models (GLM) analysis of the effect of the explanatory variables “host”, “temperature” and “sex” on the response variables “Post-embryonic development” and “adult longevity”, of Dicyphus cerastii reared on tomato, Cape gooseberry and tobacco at 15, 20 and 25°C, with factitious prey. S5 Table. Longevity in days (mean ± SE) of male and female Dicyphus cerastii at three different temperatures (15, 20, 25°C) with factitious prey. S6 Table. Generalized linear models (GLM) analysis of the effect of the explanatory variables “temperature” and “sex” on the response variables “embryonic development”, “Nymph instar”, “Post-embryonic development” and “adult longevity” of Dicyphus cerastii reared on tomato, at 15.0, 20.0, 25.0, 27.5, 30.0 and 32.5°C, with factitious prey. S7 Table. Log-Rank test comparison of Dicyphus cerastii nymph survival curves on tomato at different temperatures, with factitious prey. S8 Table. Log-Rank test comparison of Dicyphus cerastii survival curves (from egg hatch to adult death) on tomato at different temperatures, with factitious prey. S9 Table. Mean values (± SE) of parameters of three models describing the developmental rate of Dicyphus cerastii. (DOCX) [file pone.0320847.s001.docx]

Supporting information

**S1 Table.** Modelling mathematical equations used to fit temperature (T) and developmental rate (1/D) relationship.

| **Model** | **Equation** | **Calculated parameters** |
| --- | --- | --- |
| Linear | 1/D = a + bT | a,b = constants |
| Lactin-2 | 1/D = e ^(ρ T)^ – e ^(ρ TL - (TL-T)/ΔT)^ + λ | ρ = constant LT = lethal maximum temperature ΔT = temperature range over which physiological breakdown becomes the overriding influence  λ = constant |
| Brière-1 | 1/D = aT (T-T_0_) (T_L_-T) ^1/2^ | a = constant T_0_ = lower developmental threshold T_L_ = lethal threshold |

**S2** **Table.** Log-Rank test comparison of *Dicyphus cerastii* survival curves on tomato, tobacco, and Cape gooseberry at different temperatures, without factitious prey.

| **Comparison** | **χ^2^** | **d.f.** | ***p*** |
| --- | --- | --- | --- |
| 15 °C |  |  |  |
| tomato – tobacco | 46.8 | 1 | <0.001 |
| tomato – C. gooseberry | 62.3 | 1 | <0.001 |
| tobacco – C. gooseberry | 0.2 | 1 | 0.7 |
| 20.0 °C |  |  |  |
| tomato – tobacco | 29.4 | 1 | <0.001 |
| tomato – C. gooseberry | 31.8 | 1 | <0.001 |
| tobacco – C. gooseberry | 0.1 | 1 | 0.8 |
| 25.0 °C |  |  |  |
| tomato – tobacco | 14.5 | 1 | <0.001 |
| tomato – C. gooseberry | 4.9 | 1 | 0.003 |
| tobacco – C. gooseberry | 3.9 | 1 | 0.05 |
| Tomato |  |  |  |
| 15–20 °C | 2.4 | 1 | 0.1 |
| 15–25 °C | 24.9 | 1 | <0.001 |
| 20–25 °C | 8.1 | 1 | 0.004 |
| Tobacco |  |  |  |
| 15–20 °C | 5 | 1 | 0.03 |
| 15–25 °C | 16.9 | 1 | <0.001 |
| 20–25 °C | 4.5 | 1 | 0.03 |
| Cape gooseberry |  |  |  |
| 15–20 °C | 8.5 | 1 | 0.003 |
| 15–25 °C | 5.8 | 1 | 0.02 |
| 20–25 °C | 1 | 1 | 0.3 |

**S3 Table.** Log-Rank test comparison of *Dicyphus cerastii* survival curves on tomato, tobacco, and Cape gooseberry at different temperatures, with factitious prey.

| **Comparison** | **χ^2^** | **d.f.** | ***p*** |
| --- | --- | --- | --- |
| 15 °C |  |  |  |
| tomato – tobacco | 3.9 | 1 | 0.05 |
| tomato – C. gooseberry | 0.9 | 1 | 0.3 |
| tobacco – C. gooseberry | 7.5 | 1 | 0.006 |
| 20.0 °C |  |  |  |
| tomato –tobacco | 2 | 1 | 0.2 |
| tomato – C. gooseberry | 0.1 | 1 | 0.8 |
| tobacco – C. gooseberry | 7.8 | 1 | 0.005 |
| 25.0 °C |  |  |  |
| tomato – tobacco | 10.9 | 1 | 0.001 |
| tomato – C. gooseberry | 3.7 | 1 | 0.05 |
| tobacco – C. gooseberry | 1.6 | 1 | 0.2 |
| Tomato |  |  |  |
| 15–20 °C | 39.3 | 1 | <0.001 |
| 15–25 °C | 73.7 | 1 | <0.001 |
| 20–25 °C | 30.9 | 1 | <0.001 |
| Tobacco |  |  |  |
| 15–20 °C | 0.4 | 1 | 0.5 |
| 15–25 °C | 4.2 | 1 | 0.04 |
| 20–25 °C | 39.2 | 1 | <0.001 |
| Cape gooseberry |  |  |  |
| 15–20 °C | 53.4 | 1 | <0.001 |
| 15–25 °C | 68.2 | 1 | <0.001 |
| 20–25 °C | 33.9 | 1 | <0.001 |

**S4 Table**. Generalized linear models (GLM) analysis of the effect of the explanatory variables “host”, “temperature” and “sex” on the response variables “Post-embryonic development” and “adult longevity”, of *Dicyphus cerastii* reared on tomato, Cape gooseberry and tobacco at 15, 20 and 25°C, with factitious prey.

| **Explanatory variable** | **d.f.** | **F** | ***p*** |
| --- | --- | --- | --- |
| Post-embryonic development | | | |
| Host | 2 | 32.71 | <0.001 |
| Temperature | 2 | 2411.38 | <0.001 |
| Sex | 1 | 1,58 | 0.210 |
| Host x Temperature | 4 | 17.40 | <0.001 |
| Host x Sex | 2 | 2,85 | 0.060 |
| Adult longevity | | | |
| Host | 2 | 1.14 | 0.322 |
| Temperature | 2 | 135.89 | <0.001 |
| Sex | 1 | 13.53 | <0.001 |
| Host x Temperature | 4 | 2.62 | 0.035 |
| Temperature x Sex | 2 | 4.54 | 0.011 |

**S5 Table.** Longevity in days (mean ± SE) of male and female *Dicyphus cerastii* at three different temperatures (15, 20, 25°C) with factitious prey

|  | **Temperature** | | |
| --- | --- | --- | --- |
| **Sex** | **15 °C** | **20 °C** | **25 °C** |
| Male | 183.3±11.2Aa | 90.2±7.1Ba | 50.3±4.3Ca |
| Female | 136.7±9.1Ab | 78.0±5.2Ba | 39.6±2.8Cb |

Means followed by the same uppercase letter within rows, or lowercase within columns, correspond to groups among which means are not significantly different for Tukey HSD test (*p*<0.05).

**S6 Table.** Generalized linear models (GLM) analysis of the effect of the explanatory variables “temperature” and “sex” on the response variables “embryonic development”, “Nymph instar”, “Post-embryonic development” and “adult longevity” of *Dicyphus cerastii* reared on tomato, at 15.0, 20.0, 25.0, 27.5, 30.0 and 32.5°C, with factitious prey.

| **Explanatory variable** | **d.f.** | **F** | ***p*** |
| --- | --- | --- | --- |
| Embryonic development | | | |
| Temperature | 5 | 4619.7 | <0.001 |
| 1^st^ Instar | | | |
| Temperature | 4 | 212.97 | <0.001 |
| 2^nd^ Instar | | | |
| Temperature | 4 | 241.06 | <0.001 |
| 3^rd^ Instar |  |  |  |
| Temperature | 4 | 118.60 | <0.001 |
| Sex | 4 | 7.36 | 0.007 |
| 4^th^ Instar |  |  |  |
| Temperature | 4 | 189.22 | <0.001 |
| 5^th^ Instar | | | |
| Temperature | 4 | 483.69 | <0.001 |
| Sex | 1 | 4.43 | 0.037 |
| Post-embryonic development | | | |
| Temperature | 4 | 735.84 | <0.001 |
| Adult longevity |  |  |  |
| Temperature | 4 | 64.45 | <0.001 |

**S7 Table.** Log-Rank test comparison of *Dicyphus cerastii* nymph survival curves on tomato at different temperatures, with factitious prey.

| **Comparison** | **χ^2^** | **d.f.** | ***p*** | |
| --- | --- | --- | --- | --- |
| 15.0 °C – 20.0 °C | 0.6 | 1 | | 0.4 |
| 15.0 °C – 25.0 °C | 7.6 | 1 | | 0.006 |
| 15.0 ºC – 27.5 °C | 3.8 | 1 | | 0.05 |
| 15.0 °C – 30.0 °C | 18.5 | 1 | | <0.001 |
| 20.0 °C – 25.0 °C | 10.7 | 1 | | 0.001 |
| 20.0 ºC – 27.5 °C | 7.5 | 1 | | 0.006 |
| 20.0 ºC – 30.0 °C | 25.9 | 1 | | <0.001 |
| 25.0 °C – 27.5 °C | 0.9 | 1 | | 0.4 |
| 25.0 °C – 30.0 °C | 2.2 | 1 | | 0.1 |
| 27.5 ºC – 30.0 °C | 6.1 | 1 | | 0.01 |

**S8 Table.** Log-Rank test comparison of *Dicyphus cerastii* survival curves (from egg hatch to adult death) on tomato at different temperatures, with factitious prey.

| **Comparison** | **χ^2^** | **d.f.** | ***p*** |
| --- | --- | --- | --- |
| 15.0 °C – 20.0 °C | 30.5 | 1 | <0.001 |
| 15.0 °C – 25.0 °C | 63.9 | 1 | <0.001 |
| 15.0 °C –27.5 °C | 71.2 | 1 | <0.001 |
| 15.0 °C – 30.0 °C | 74.2 | 1 | <0.001 |
| 20.0 ºC – 25.0 °C | 26.9 | 1 | <0.001 |
| 20.0 °C – 27.5 °C | 65.1 | 1 | <0.001 |
| 20.0 ºC – 30.0 °C | 71.7 | 1 | <0.001 |
| 25.0 °C – 27.5 °C | 20.1 | 1 | <0.001 |
| 25.0 ºC – 30.0 °C | 23.8 | 1 | <0.001 |
| 27.5 °C – 30.0 °C | 0.8 | 1 | 0.4 |

**S9 Table.** Mean values (± SE) of parameters of three models describing the developmental rate of *Dicyphus cerastii.*

| **Model** | **Parameters** | **Egg** | **N1** | **N2** | **N3** | **N4** | **N5** | **Post-embryonic** | **Total** |
| --- | --- | --- | --- | --- | --- | --- | --- | --- | --- |
| Linear | a | -0.0265±0.0150 | -0.0133±0.0455 | -0.0086±0.0930 | -0.0589±0,0631 | -0.0310±0.0367 | -0.0597±0.0210 | -0.0120±0,0068 | -0.0107±0,0038 |
|  | b | 0.0043±0.0006 | 0.0110±0.0018 | 0.0136±0.0036 | 0.0157±0,0026 | 0.0119±0.0015 | 0.0091±0.0009 | 0.0025±0,0003 | 0.0017±0,0002 |
|  | K | 230.36 | 91.17 | 73.80 | 63.60 | 84.13 | 109.47 | 393.98 | 584.76 |
|  | t_min_ | 6.1 | 1.2 | 0.6 | 3.7 | 2.6 | 6.5 | 4.7 | 6.3 |
|  | R^2^ | 0.9328 | 0.9056 | 0.7780 | 0.9232 | 0.9530 | 0.9733 | 0.9639 | 0.9749 |
|  | RSS | 0.000289 | 0.002666 | 0.011140 | 0.002982 | 0.001009 | 0.000331 | 0.000035 | 0.000011 |
|  | AIC | -36.6256 | -23.2861 | -14.7078 | -16.9332 | -22.3496 | -27.9205 | -39.1676 | -44.9791 |
| Lactin-2 | ρ | 0.0048±0.0003 | 0.0101±0.0015 | 0.0153±0.0023 | 0.0043±0.0015 | 0.0113±0.0005 | 0.0079±0.0005 | 0.0027±0.0003 | 0.0018±0.0001 |
|  | T_L_ (T_max_) | 38.2±0.4 | 36.5±0.5 | 37.3±0.6 | 34.0±0.4 | 34.2±0.2 | 33.5±0.6 | 35.2±0.7 | 37.7±0.5 |
|  | ∆T | 1.5±0.2 | 1.2±0.4 | 2.2±0.6 | 1.3±0.3 | 1.3±0.1 | 0.6±0.4 | 1.0±0.3 | 1.0±0.2 |
|  | λ | -1.04±0.01 | -1.02±0.05 | -1.09±0.07 | -1.07±0.04 | -1.05±0.01 | -1.05±0.01 | -1.02±0.01 | -1.01±0.00 |
|  | t_min_ | 8.4 | 1.8 | 5.9 | 5.0 | 4.0 | 5.9 | 6.0 | 7.5 |
|  | t_opt_ | 30.8 | 31.2 | 29.6 | 28.6 | 28.7 | 30.3 | 29.2 | 29.2 |
|  | R^2^ | 0.9957 | 0.9778 | 0.9787 | 0.9928 | 0.9987 | 0.9972 | 0.9943 | 0.9980 |
|  | RSS | 0.000043 | 0.001926 | 0.003054 | 0.000855 | 0.000093 | 0.000092 | 0.000016 | 0.000002 |
|  | AIC | -54.1302 | -27.5214 | -24.2942 | -26.1072 | -39.4525 | -39.5058 | -49.84 | -61.48 |
| Brière-1 | a | 0.00006±0.00000 | 0.00009±0.00006 | 0.00023±0.00004 | 0.00020±0.00008 | 0.00015±0.00001 | 0.00003±0.00004 | 0.00002±0.00001 | 0.00002±0.00001 |
|  | t_min_ (T_0_) | 6.9±0.6 | -4.7±12.9 | 4.8±2.8 | 3.3±5.3 | 1.6±0.7 | -7.8±16.0 | -1.8±7.0 | 3.9±3.6 |
|  | t_max_ (T_L_) | 37.4±0.4 | 40.8±5.8 | 35.8±0.9 | 35.8±2.9 | 36.0±0.3 | 61.3±46.4 | 40.8±5.8 | 38.1±3.3 |
|  | t_opt_ | 30.6 | 32.2 | 29.2 | 29.0 | 29.0 | 48.4 | 32.4 | 30.8 |
|  | R^2^ | 0.9986 | 0.9223 | 0.9223 | 0.9668 | 0.9996 | 0.9922 | 0.9875 | 0.9888 |
|  | RSS | 0.000006 | 0.001835 | 0.001479 | 0.001290 | 0.000008 | 0.000097 | 0.000011 | 0.000005 |
|  | AIC | -58.0480 | -23.5260 | -24.8233 | -19.1219 | -44.3218 | -32.0656 | -42.8202 | -47.0284 |
